# Volume electron microscopy of the distribution of synapses in the neuropil of the juvenile rat somatosensory cortex

**DOI:** 10.1007/s00429-017-1470-7

**Published:** 2017-07-18

**Authors:** A. Santuy, J. R. Rodriguez, J. DeFelipe, A. Merchan-Perez

**Affiliations:** 1 Laboratorio Cajal de Circuitos Corticales, Centro de Tecnología Biomédica, Universidad Politécnica de Madrid, Pozuelo de Alarcón, 28223 Madrid, Spain; 2 Instituto Cajal, Consejo Superior de Investigaciones Científicas, 28002 Madrid, Spain; 3 CIBERNED, Centro de Investigación Biomédica en Red de Enfermedades Neurodegenerativas, Madrid, Spain; 4 Departamento de Arquitectura y Tecnología de sistemas Informáticos, Universidad Politécnica de Madrid, Boadilla del Monte, 28660 Madrid, Spain

**Keywords:** Excitatory synapses, Dendritic shafts, Somatosensory cortex, Dendritic spines, Inhibitory synapses, Serial section three-dimensional reconstruction, Dual-beam electron microscopy, FIB/SEM

## Abstract

Knowing the proportions of asymmetric (excitatory) and symmetric (inhibitory) synapses in the neuropil is critical for understanding the design of cortical circuits. We used focused ion beam milling and scanning electron microscopy (FIB/SEM) to obtain stacks of serial sections from the six layers of the juvenile rat (postnatal day 14) somatosensory cortex (hindlimb representation). We segmented in three-dimensions 6184 synaptic junctions and determined whether they were established on dendritic spines or dendritic shafts. Of all these synapses, 87–94% were asymmetric and 6–13% were symmetric. Asymmetric synapses were preferentially located on dendritic spines in all layers (80–91%) while symmetric synapses were mainly located on dendritic shafts (62–86%). Furthermore, we found that less than 6% of the dendritic spines establish more than one synapse. The vast majority of axospinous synapses were established on the spine head. Synapses on the spine neck were scarce, although they were more common when the dendritic spine established multiple synapses. This study provides a new large quantitative dataset that may contribute not only to the knowledge of the ultrastructure of the cortex, but also towards defining the connectivity patterns through all cortical layers.

**Electronic supplementary material:**

The online version of this article (doi:10.1007/s00429-017-1470-7) contains supplementary material, which is available to authorized users.

## Introduction

The cerebral cortex displays a highly complex synaptic organization but there are general rules that can be applied—albeit with a certain degree of variation—to all the cortical areas and species examined (DeFelipe 2011). Most cortical synapses (90–98%) are established in the neuropil (Alonso-Nanclares et al. 2008), which is composed of dendrites, axons and glial processes. There are two main morphological types of synapses that can be identified based on morphological criteria—asymmetric and symmetric synapses (Colonnier 1968; Gray 1959). This distinction is important because, in general, asymmetric synapses (AS) are excitatory (glutamatergic) and symmetric synapses (SS) are inhibitory (GABAergic) (Ascoli et al. 2008). Although it has been described that terminals that establish AS and SS can synthesize neurotransmitters other than glutamate and GABA, respectively, such as acetylcholine, serotonin, noradrenaline or dopamine, a large proportion of these axonal systems are non-synaptic (Beaulieu and Somogyi 1990; DeFelipe and Jones 1988; Descarries and Mechawar 2000). Therefore, it follows that AS and SS that use acetylcholine, serotonin, noradrenaline or dopamine must represent a very small proportion of the total number of AS and SS. Synapses can be established on dendritic spines (for simplicity, spines) which are mostly present in excitatory neurons, or on dendritic shafts of either excitatory or inhibitory neurons. Excitatory synapses are the most abundant (80–90% of all cortical synapses) and originate from cortical spiny neurons and extrinsic cortical afferents, while inhibitory synapses are less numerous (about 10–20%) and mainly originate from local interneurons (Beaulieu and Colonnier 1985; DeFelipe 2011; DeFelipe and Fariñas 1992; Feldman 1984; Silberberg 2008; White 2007; White and Keller 1989). Thus, knowing the proportions of AS and SS synapses and their distribution on spines and dendritic shafts is critical for understanding the design of cortical circuits. Of particular interest is the study of spines. These structures were described for the first time by Cajal in 1888 (DeFelipe 2015) and since then spines have been studied using multiple techniques as they are thought to be key elements in memory, learning and cognition (Yuste 2010). In general, it is assumed that the majority of excitatory synapses are established on spines with a ratio of one spine/one synapse. However, it has been reported that some spines lack synapses, while other spines can have multiple synapses (Feldman 1984; Harris et al. 1992; Harris and Kater 1994; Jones and Powell 1969; Popov et al. 2005).

The identification of AS and SS and the proportions of these synapses in the neuropil is relatively easy, using conventional electron microscope techniques (DeFelipe et al. 1999) as it does not require the examination of long series of sections, which is a major limitation of conventional electron microscopy. However, the identification of the postsynaptic structure (i.e., whether the synapse is established on a spine or on a dendritic shaft) often requires the examination of several consecutive sections. In addition, determining whether spines receive one or more synapses and the morphological type of these axospinous synapses are major challenges. Overcoming such challenges requires the use of electron microscopy with serial section 3D reconstruction. Here is where automated or semi-automated electron microscopy techniques come into play (Denk and Horstmann 2004; Helmstaedter 2013; Knott et al. 2008; Merchan-Perez et al. 2009; Morgan and Lichtman 2013; Smith 2007)—techniques that involve much less-demanding human interaction and training than for conventional electron microcopy.

Here, we use a dual-beam electron microscope that combines a focused ion beam (FIB) column that mills the sample and a scanning electron microscope (SEM) that images the freshly exposed surface. Combining both beams sequentially, we obtain long series of images that represent three-dimensional samples of tissue. This technique has been used before for the study of synapses and several software packages have been developed to analyze the images obtained (Knott et al. 2008; Kreshuk et al. 2011; Merchan-Perez et al. 2009, 2014; Morales et al. 2011, 2013). In this work, we have studied the proportions of AS and SS on spines and dendritic shafts in the neuropil of all cortical layers of the somatosensory cortex of Wistar rats on postnatal day 14 (P14). We have used P14 rats with the aim of integrating the present data with other anatomical, molecular and physiological data that have been obtained in the same cortical region of P14 rats, to create biologically accurate large-scale cortical models (Markram et al. 2015). The densities of the different types of synapses have been quantified across all cortical layers, as well as the occurrence of single or multiple synapses on the same spine.

## Materials and methods

### Tissue preparation

Three male Wistar rats killed on postnatal day 14 were used for this study. Animals were administered a lethal intraperitoneal injection of sodium pentobarbital (40 mg/kg) and were intracardially perfused with 2% paraformaldehyde and 2.5% glutaraldehyde in 0.1 M phosphate buffer (PB). The brain was then extracted from the skull and processed for electron microscopy as previously described (Merchan-Perez et al. 2009). Briefly, the brains were extracted from the skull and post-fixed at 4 °C overnight in the same solution used for perfusion. They were then washed in PB and vibratome sections (150 μm thick) were obtained. Sections containing the primary somatosensory cortex (hindlimb representation) were selected with the help of an atlas (Paxinos and Watson 2007). Selected sections were osmicated for 1 h at room temperature in PB with 1% OsO4, 7% glucose and 0.02 M CaCl_2_. After washing in PB, the sections were stained for 30 min with 1% uranyl acetate in 50% ethanol at 37 °C, and they were then dehydrated and flat embedded in Araldite (DeFelipe and Fairen 1993). Embedded sections were glued onto blank Araldite stubs and trimmed. To select the exact location of the samples, we first obtained plastic semithin sections (1–2 μm thick) from the block surface and stained them with toluidine blue to identify cortical layers. These sections were then photographed with a light microscope. The last of these light microscope images (corresponding to the section immediately adjacent to the block face) was then collated with SEM photographs of the surface of the block. In this way, it was possible to accurately identify the regions of the neuropil to be studied.

All animals were handled in accordance with the guidelines for animal research set out in the European Community Directive 2010/63/EU, and all procedures were approved by the local ethics committee of the Spanish National Research Council (CSIC).

### Three-dimensional electron microscopy

Three-dimensional brain tissue samples of the somatosensory cortex (hindlimb representation) were obtained using combined focused ion beam milling and scanning electron microscopy (FIB/SEM). We focused on the neuropil, which is composed of axons, dendrites and glial processes, so the samples did not contain cell somata, proximal dendrites in the immediate vicinity of the soma, or blood vessels.

We used a Neon40 EsB electron microscope (Carl Zeiss NTS GmbH, Oberkochen, Germany). This instrument combines a high-resolution field emission SEM column with a focused gallium ion beam, which can mill the sample surface, removing thin layers of material on a nanometer scale. After removing each slice (20 nm thick), the milling process was paused, and the freshly exposed surface was imaged with a 1.8-kV acceleration potential using the in-column energy selective backscattered (EsB) electron detector. The milling and imaging processes were sequentially repeated, and long series of images were acquired through a fully automated procedure, thus obtaining a stack of images that represented a three-dimensional sample of the tissue (Merchan-Perez et al. 2009). Twenty nine different samples (stacks of images) of the neuropil of the six layers of the somatosensory cortex were obtained (three samples of layer I, four of layer II, ten of layer III, five of layer IV, three of layer V and four of layer VI (see Supplementary Table 1 in Online Resource 1). Most of these image stacks have been used previously for the study of the spatial distribution of synapses. In these previous studies, we estimated the global density of synapses in the neuropil using twenty five image stacks of the six layers of the rat somatosensory cortex (Anton-Sanchez et al. 2014; Merchan-Perez et al. 2014). In the present work, we have added four more image stacks (one for layer I, one for layer II, two for layer IV) and we have studied in detail the densities of asymmetric and symmetric synaptic junctions. To estimate the density of synapses in each stack, we counted the number of synaptic junctions within an unbiased three-dimensional counting frame of known volume (Howard and Reed 2005). Image resolution in the xy plane ranged from 3.7 to 4.5 nm/pixel. Resolution in the z axis (section thickness) was 20 nm and image sizes were 2048 × 1536 pixels. Better resolutions can be achieved but it must be considered that, for example, doubling the resolution would result in a field of view that is one-fourth of the original surface. Therefore, we chose these resolution parameters as a compromise between the resolution and the field of view that still allowed us to identify different types of synapses. The number of sections per stack ranged from 189 to 363 (mean 254.66; total 7385 sections). The volumes of the stacks, after correction for tissue shrinkage (Merchan-Perez et al. 2009), ranged from 225.13 to 508.96 μm^3^ (mean 306.55 μm^3^; total 8889.82 μm^3^). The volumes of the counting frames ranged from 123.81 to 280.09 μm^3^ (mean = 181.62 μm^3^; total 5266.88 μm^3^) (see Supplementary Table 1 in Online Resource 1).

### Identification of synapses and their postsynaptic targets

Synaptic junctions within these volumes were visualized and segmented in 3D with Espina software (Morales et al. 2011). The segmentation algorithm makes use of the fact that presynaptic and postsynaptic densities appear as dark, electron-dense structures under the electron microscope. It requires a Gaussian blur filter preprocessing step to eliminate noisy pixels followed by a gray-level threshold to extract all the voxels that fit the gray levels of the synaptic junction. In this way, the resulting 3D segmentation includes both the pre- and post-synaptic densities (Morales et al. 2013). As previously described, synaptic junctions with a prominent or thin PSD were classified as asymmetric or symmetric synaptic junctions, respectively (Merchan-Perez et al. 2009). To facilitate this task, especially when synaptic junctions were cut obliquely or “en face”, the stacks of serial images were digitally resliced through orthogonal planes of section, so the synaptic junctions could be visualized from different perspectives, as previously described (Merchan-Perez et al. 2009; Morales et al. 2011). To identify the postsynaptic targets of these synapses, we navigated the image stack to determine whether the postsynaptic element was a spine or a dendritic shaft as shown in the video (Online Resource 2). Unambiguous identification of spines requires that the spine must be visually traced to the parent dendrite. Similarly, unambiguous identification of dendritic shafts, especially if they are thin, requires that they can be visually followed inside the stack over a long enough path. Accordingly, when the postsynaptic element of a synapse was close to the margins and it was truncated by the borders of the stack, the identity of the postsynaptic target could not be determined. Therefore, the targets of synapses in any of the stacks were classified into two main categories: spines and dendritic shafts, while truncated elements that could not be safely identified were labeled as “truncated”. When the postsynaptic target was a spine, we further recorded the position of the synapse on the head or neck. We also recorded the presence of single or multiple synapses on a single spine.

We also determined whether the target dendrite was a spiny dendrite or a non-spiny one. If a given dendrite had spines, it was considered as belonging to a pyramidal neuron, except in layer IV where spines may belong to pyramidal cells, spiny stellate cells or other types of spiny non-pyramidal cells. However, if a dendritic segment did not have spines, it was classified as “not determined”, since it could either belong to a non-spiny neuron (most probably an interneuron) or to a dendritic segment from a spiny neuron that does not have spines in the volume of tissue examined.

### Statistical analysis

To study whether there were significant differences between synaptic distributions among the different layers, we performed a multiple mean comparison test on the 29 samples of the six cortical layers. Since the necessary assumptions for ANOVA were not satisfied (the normality and homoscedasticity criteria were not met), we used the Kruskal–Wallis test (KW) and the Mann–Whitney test (MW) for pair-wise comparisons. *χ* ^2^ tests were used for contingency tables.

## Results

### Distribution of synapses on spines and shafts

**Fig. 1:**
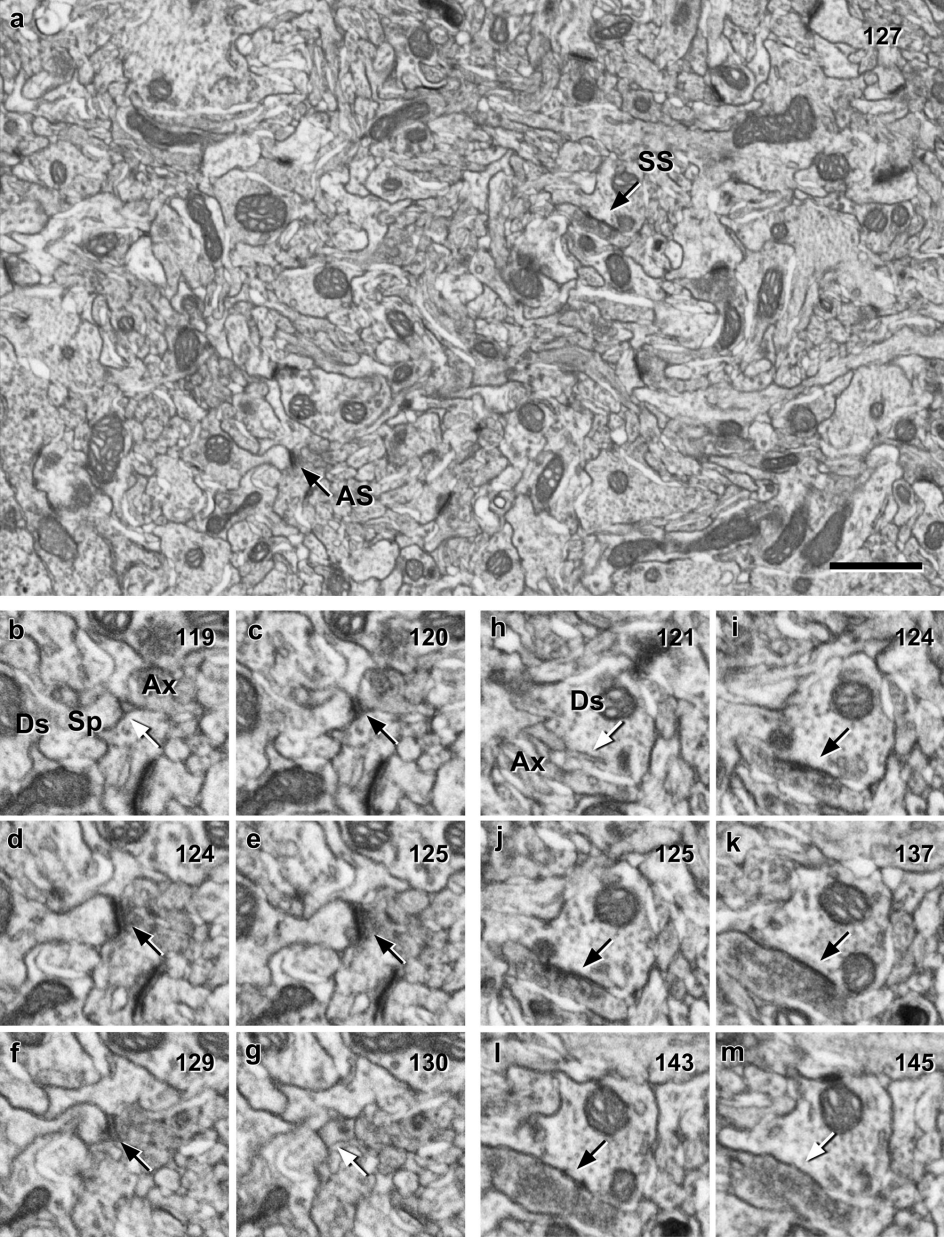
Serial images obtained with combined focused ion beam milling and scanning electron microcopy (FIB/SEM). In this example, 275 serial images were taken at a resolution of 5 nm/pixel in the *X* and *Y* axes, and a milling depth (section thickness) of 20 nm. **a** Low magnification image of section nr. 127 where numerous synaptic junctions can be identified. Two of these synapses have been selected as examples (*black arrows*) of asymmetric (AS) and symmetric (SS) synapses. AS had a prominent postsynaptic density, while SS showed a thin postsynaptic density, very similar to the presynaptic density. Note that these classifications were not based on single images but on the examination of the full sequence of images where each synapse was visible (numbers in the top-right corner of each frame correspond to section number). For example, AS is not yet visible in (**b**) (*white arrow*), but it can be visualized in (**c**–**f**) (*black arrows*), until it disappears again in (**g**) (*white arrow*). Similarly, SS is not yet visible in (**h**) (*white arrow*), it can be identified in (**i**) to (**l**) (*black arrows*) and it has disappeared in (**m**) (*white arrow*). Navigating the stack of serial sections also helps to identify the postsynaptic target of AS as a spine and the postsynaptic target of SS as a dendritic shaft. *Ax* Axon, *Ds* dendritic shaft, *Sp* spine. *Scale bar* in (**a**) indicates 1 µm in (**a**) and 556 nm in (**b**–**m**)

**Fig. 2:**
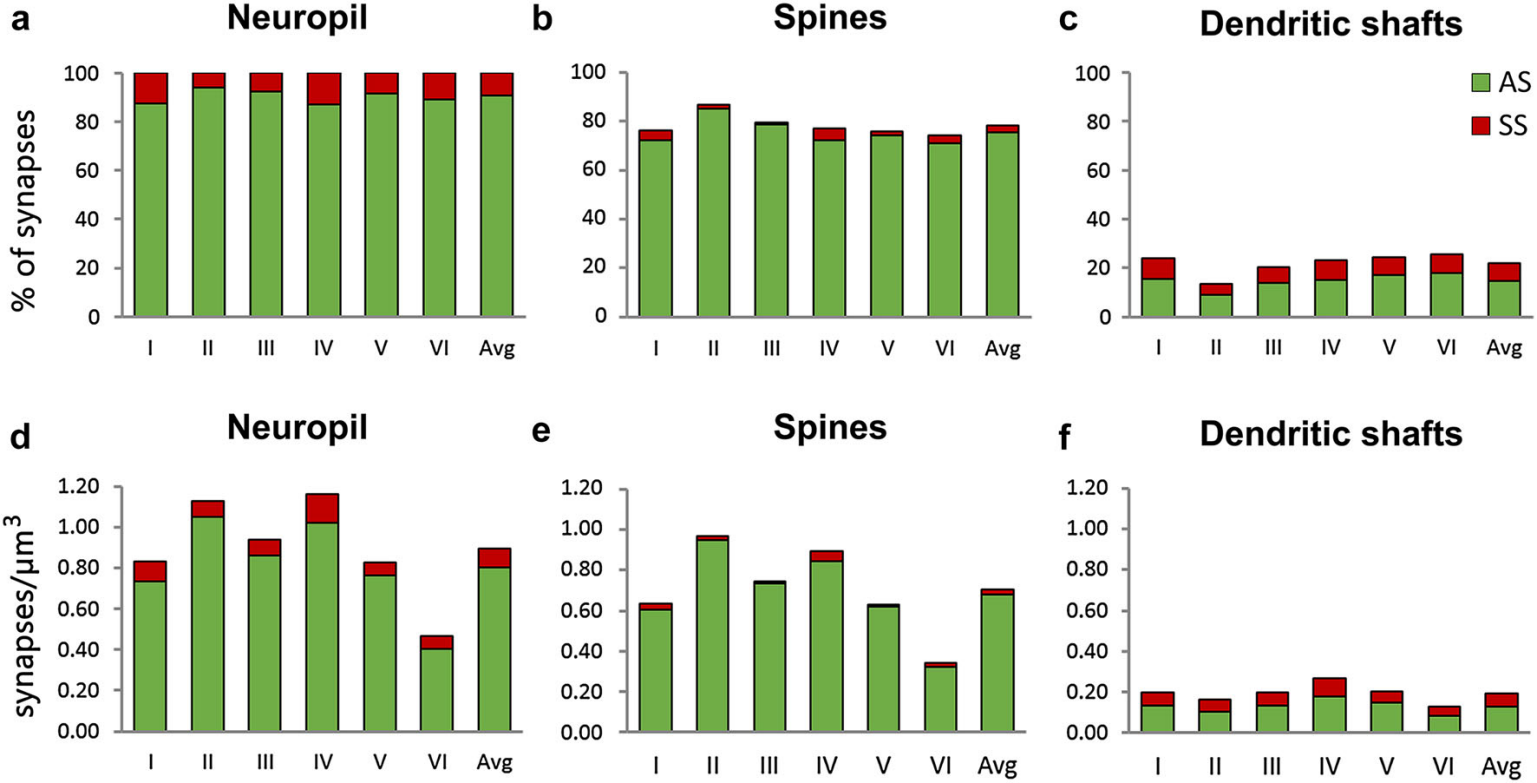
Proportions of asymmetric (AS) and symmetric (SS) synapses in the neuropil and their distribution on spines and dendritic shafts. **a** Percentage of AS (*green*) and SS (*red*) in the neuropil of the six cortical layers and as an average (Avg). The percentages of AS and SS on spines and dendritic shafts are shown in (**b**) and (**c**), respectively. AS on spines predominate in all layers, followed by AS on dendritic shafts, SS on dendritic shafts and SS on spines. Statistically significant differences were only found between AS on spines of layers II and VI (MW, *p* < 0.05). **d** Density of AS (*green*) and SS (*red*) in the neuropil of the six layers of the cortex and as an average (Avg), measured as the number of synaptic junctions per µm^3^. The densities of AS and SS on spines and dendritic shafts are shown in (**e**) and (**f**), respectively. A significantly lower density of synapses was found in layer VI for both AS and SS (MW, *p* < 0.05). (See also Table 1)

In our samples, we identified a total number of 7567 synaptic junctions in all cortical layers. Of these, we discarded 1383 (18.28%) because the structures onto which synapses were established were truncated by the margins of the stack so it was not possible to identify with certainty if these postsynaptic structures were dendritic spines or dendritic shafts. Thus, we finally analyzed 6184 synapses whose targets were unambiguously identified as spines or dendritic shafts (Fig. 1; see also Video in Online Resource 2). The proportion of AS in our samples ranged from 87.29% in layer IV to 94.11% in layer II (mean 90.28%). Thus, for SS, the proportions ranged from 12.71% in layer IV to 5.89% in layer II (mean 9.72%) (Table 1; Fig. 2a). Although the proportions of AS and SS varied between layers, differences were not significant (KW, *p* = 0.05). No differences were found either when we compared supragranular layers (considering layers I, II and III together) with granular and infragranular layers (layers IV, V and VI considered together) (KW, *p* = 0.08).

**Table 1:** Distribution of asymmetric (AS) and symmetric synapses (SS) on spines and dendritic shafts

	Layer						
	I	II	III	IV	V	VI	Average (I–VI)
AS on dendritic spine heads	**70.95**	**84.09**	**76.51**	**69.93**	**72.71**	**69.27**	**73.91%**
	(425)	(814)	(1671)	(858)	(477)	(381)	
AS on dendritic spine necks	**1.17**	**1.14**	**1.97**	**2.20**	**1.52**	**1.82**	**1.64%**
	(7)	(11)	(43)	(27)	(10)	(10)	
AS on dendritic shafts	**15.36**	**8.88**	**13.74**	**15.16**	**17.23**	**18.00**	**14.73%**
	(92)	(86)	(300)	(186)	(113)	(99)	
SS on dendritic spine heads	**2.50**	**0.72**	**0.82**	**4.24**	**0.91**	**2.55**	**1.96%**
	(15)	(7)	(18)	(52)	(6)	(14)	
SS on dendritic spine necks	**1.50**	**0.62**	**0.27**	**0.57**	**0.46**	**0.73**	**0.69%**
	(9)	(6)	(6)	(7)	(3)	(4)	
SS on dendritic shafts	**8.51**	**4.55**	**6.68**	**7.91**	**7.16**	**7.64**	**7.08%**
	(51)	(44)	(146)	(97)	(47)	(42)	
Total	**100**	**100**	**100**	**100**	**100**	**100**	
	(599)	(968)	(2184)	(1227)	(656)	(550)	

Synapses on spines have been sub-divided into those that are established on spine heads and those established on spine necks. Data are given in percentages (bold typeface) and absolute numbers of synapses studied (parentheses) for each of the six cortical layers (I–VI). Average values as a percentage for all layers are given in the column on the right

**Fig. 3:**
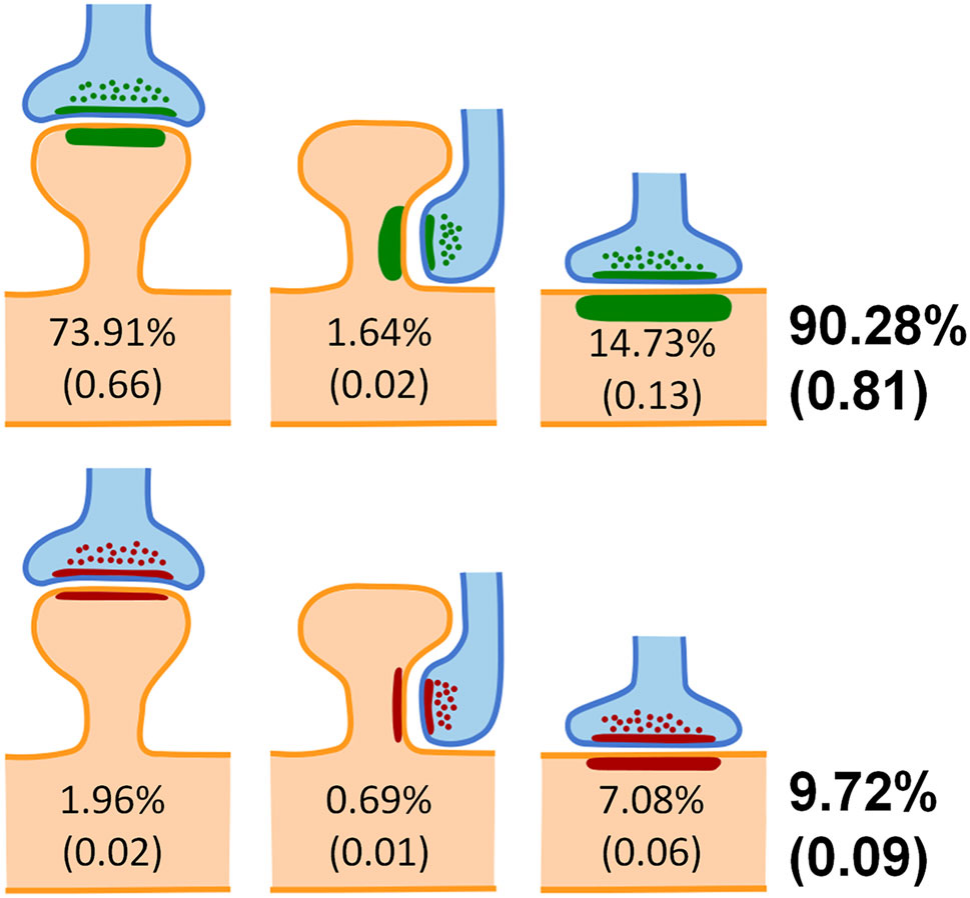
Schematic representation of the distribution of asymmetric synapses (*green*) and symmetric synapses (*red*) on spines and dendritic shafts. Synapses on spines have been sub-classified into those that are established on the head of the spine and those that are established on the neck. Percentages represent the average of the six cortical layers. Values between parentheses represent the density of each type of synapse in the neuropil, in synapses per µm^3^

Analysis of the preferred target showed that the proportion of synapses established on spines ranged from 74.36% in layer VI to 86.57% in layer II (mean 78.20%). The remaining synapses (mean 21.80%) were established on dendritic shafts. For AS, the proportion of synapses established on spines ranged from 79.80% in layer VI to 90.56% in layer II (mean 83.61%). The remaining AS (mean 16.40%) were established on dendritic shafts. This contrasts with the distribution of SS; in this case a higher proportion of synapses were found on dendritic shafts (mean 74.53%), ranging from 62.18% in layer IV to 85.88% in layer III. About a quarter of SS were established on spines (25.47%) (Table 1; Figs. 2b, c, 3). We further analyzed the possible differences between cortical layers regarding the proportions of AS and SS synapses and their targets. Statistically significant differences were only found in the distribution of AS on spines and shafts between layers II—where the proportion of AS on spines was the highest—and VI—where the proportion of AS on spines was the lowest, (MW, *p* < 0.05) as shown in Fig. 2b, c. When we focused on the location of axospinous synapses, we found that most of them (97.02%) were located on the spine head, while just 2.98% were located on the neck (Table 1; Fig. 3).

In summary, most synapses in the neuropil were axospinous AS, followed by AS established on dendritic shafts, SS on dendritic shafts and axospinous SS (Table 1; Figs. 2, 3). Given that AS outnumber SS approximately 9:1, and also axospinous synapses outnumber synapses on shafts about 8:2, it could be argued that this distribution could merely arise by chance. To rule out this possibility, 2 × 2 contingency tables were created showing both types of synapses against the type of their postsynaptic target in each cortical layer (Table 2 and Supplementary Table 2 in Online Resource 1). In these tables, the expected counts of AS and SS on spines and shafts are calculated from the marginal totals, assuming the null hypothesis that there is no association between the type of synapse and the type of postsynaptic target. In other words, the null hypothesis assumes that AS and SS have no preference for spines or shafts. In general, for any contingency table, the expected frequency for a cell in the *i*th row and the *j*th column is $E_{ij} = T_{i} T_{j} /T$ where *T*_*i*_ is the marginal total for the *i*th row, *T*_*j*_ is the marginal total for the *j*th column, and *T* is the total number of observations. *χ* ^2^ tests of association were applied to these tables. Results indicated that the null hypothesis—the type of synapse is not associated to the type of postsynaptic target—must be rejected in all cases (*p* < 0.0001). Indeed, if SS were distributed at random between spines and shafts they would be more frequent on spines than on shafts, but the opposite occurs. For example, in layer II the expected number of SS established on dendritic shafts under the null hypothesis would be 7.65 (57 × 130/968, see Table 2). However, the actual count is 44, that is, 5.75 times higher than expected. In fact, the proportion of SS established on dendritic shafts was always much higher than expected by chance (from 2.70 times higher in layer IV to 5.75 times higher in layer II; see Table 2 and Supplementary Table 2 in Online Resource 1). This suggests that SS do have a preference for dendritic shafts, but no assumption is made about the possible underlying mechanisms. Finally, the proportion of AS that established axospinous synapses was only 1.05–1.08 times higher than what would be expected by chance (Table 2 and Supplementary Table 2 in Online Resource 1).

**Table 2:** Two examples of contingency tables showing the type of synapse against the type of postsynaptic target in cortical layers II and IV

**Layer II**		Type of postsynaptic target		
		Spine	Shaft	Totals
Type of synapse	AS	**825**	**86**	911
		(788.65)	(122.35)	
	SS	**13**	**44**	57
		(49.35)	(7.65)	
	Totals	838	130	968

**Layer IV**		Type of postsynaptic target		
		Spine	Shaft	Totals
Type of synapse	AS	**885**	**186**	1071
		(823.98)	(247.02)	
	SS	**59**	**97**	156
		(120.02)	(35.98)	
	Totals	944	283	1227

The observed counts of synapses in each subcategory were taken from Table 1 and are shown in bold. The expected counts (in parentheses) are calculated from the marginal totals, assuming the null hypothesis that there is no association between the type of synapse and the type of postsynaptic target (see text for details). *χ* ^2^ tests of association were applied to these tables indicating that the null hypothesis must be rejected in all cases (*p* < 0.0001). For example, in layer II the number of SS established on dendritic shafts (44) is 5.75 times higher than would be expected by chance (7.65); in layer IV, the number of SS established on dendritic shafts (97) is 2.70 times higher than would be expected by chance (35.98). See the rest of the layers in Supplementary Table 2 in Online Resource 1

### Distribution of synapses on dendritic shafts of spiny and aspiny dendritic segments

As stated above, 21.80% of synapses were established on dendritic shafts. We were further interested in knowing whether dendritic shafts that established synapses belonged to spiny neurons (mainly pyramidal and spiny stellate cells) or to aspiny neurons (mainly interneurons). Thus, once a synapse is identified on a dendritic shaft we need to follow the dendritic segment to ascertain whether it has spines. If spines are found, we can conclude that it belongs to a spiny neuron. However, if we do not find spines, we cannot conclude that it belongs to an aspiny neuron, since it could simply belong to a dendritic segment of a spiny neuron that does not have any spines within the volume of neuropil studied. We found that at least 58.98% of synapses on shafts (12.86% of all synapses) belonged to spiny dendrites. The percentage of synapses established on shafts of spiny dendrites was slightly higher for AS (59.93%) than for SS (56.90%), although this difference was not statistically significant (*χ* ^2^ test, *p* = 0.30). In the remaining 41.02% of the synapses that were established on shafts (8.94% of all synapses), the dendritic segments did not have any spine in the volume of tissue studied. We must consider, as mentioned above, that this population of apparently smooth dendrites may contain some unnoticed spiny dendrites. Therefore, given that 78.20% of synapses are axospinuous and 12.86% are established on spiny shafts, we can conclude that *at least* 91.06% of synapses are established on spiny neurons. The exact percentage of synapses that are established on truly aspiny neurons cannot be unambiguously determined with our present methodology, but in any case it would be equal to or lower than 8.94%.

### Density of AS and SS on spines and dendritic shafts

To estimate the density of synapses in each stack of images, we counted the number of synaptic junctions within an unbiased three-dimensional counting frame of known volume (see Methods). Total synaptic densities (AS+SS) and densities of AS were highest in layers II and IV and reached their lowest value in layer VI (Fig. 2d; Table 3). Pair-wise tests confirmed that the total density of synapses and the density of AS were lower in the neuropil of layer VI than in layers II and IV (MW, *p* < 0.05). Regarding SS, their density was highest in layer IV and again lowest in layer VI; differences between layer IV and layer VI were statistically significant (MW, *p* < 0.05) (Fig. 2d; Table 3).

**Table 3:** Densities of synapses in the neuropil

	Layer						
	I	II	III	IV	V	VI	Avg
Density of all synapses (AS+SS)	0.83	1.13	0.94	1.16	0.83	0.47	0.89
Percent synapses on spines	76.13	86.57	79.58	76.94	75.61	74.36	78.20
Percent synapses on shafts	23.87	13.43	20.42	23.06	24.39	25.64	21.80
Density of synapses on spines	0.63	0.98	0.75	0.89	0.63	0.35	0.70
Density of synapses on dendritic shafts	0.20	0.15	0.19	0.27	0.20	0.12	0.19
Density of AS synapses	0.73	1.05	0.86	1.02	0.76	0.40	0.81
Percent AS on spines	82.44	90.56	85.10	82.63	81.17	79.80	83.62
Percent AS on shafts	17.56	9.44	14.90	17.37	18.83	20.20	16.38
Density of AS on spines	0.61	0.95	0.73	0.84	0.62	0.32	0.68
Density of AS on dendritic shafts	0.13	0.10	0.13	0.18	0.14	0.08	0.13
Density of SS synapses	0.10	0.08	0.08	0.14	0.07	0.06	0.09
Percent SS on spines	32.00	22.81	14.12	37.82	16.07	30.00	25.47
Percent SS on shafts	68.00	77.19	85.88	62.18	83.93	70.00	74.53
Density of SS on spines	0.03	0.02	0.01	0.05	0.01	0.02	0.02
Density of SS on dendritic shafts	0.07	0.06	0.07	0.09	0.06	0.04	0.06

Density of synapses (as no. of synapses/μm^3^) and percentage of asymmetric (AS) and symmetric synapses (SS) on spines and dendritic shafts in the neuropil of the six cortical layers (I–VI). Average values (Avg) are given in the column on the right. See also Supplementary Table 1 in Online Resource 1

We have calculated the densities of AS and SS on spines and shafts from the unbiased estimates of the densities of AS and SS in the different layers and from the proportions of synapses established on spines and dendritic shafts (Fig. 2e, f; Table 3). The density of AS on spines was highest in layer II and IV and lowest in layer VI (MW, *p* < 0.05). The density of AS located on shafts was higher in layer IV compared to layer II and VI (MW, *p* < 0.05). Regarding SS on spines, the highest density was found in layer IV and the lowest was found in layers III and V (MW, *p* < 0.05). No differences between layers were found for SS on dendritic shafts (KW, *p* = 0.11).

### Spines with multiple synapses

In our sample, 94.43% of the spines established one synapse and the remaining 5.57% established two or three synapses. We found no cases in which there were more than three synapses on the same spine (Table 4).

**Table 4:** Spines establishing single and multiple synapses

	Count	%
Spines with a single synapse	4304	94.43
One AS	4246	98.65
One SS	58	1.35
Spines with multiple synapses	254	5.57
Two AS	146	57.48
One AS and one SS	84	33.07
Two AS and one SS	9	3.54
Three AS	6	2.36
Two SS	6	2.36
Three SS	2	0.79
One AS and two SS	1	0.39

94.43% of the total population of spines studied established a single synapse. The remaining 5.57% established multiple synapses. For the latter population, the percentages of the different combinations of multiple synapses are also detailed. AS—asymmetric synapse; SS—symmetric synapse

When spines formed only one synapse, in 98.65% of the cases this was an AS, and only in 1.35% of the spines the synapse was a SS. When multiple synapses occurred on the same spine, in 79.28% of the cases they were AS, while SS accounted for the remaining 20.72%. Spines with two AS were most common (57.48%), followed by spines establishing one AS and one SS (33.07%). Spines with other combinations of AS and SS were much less abundant, representing less than 10% of the total number of spines with multiple synapses (Table 4). From a different perspective, about two-thirds (63.39%) of the total number of spines established more AS than SS, almost a third (33.07%) had the same number of AS as SS, while spines with more SS than AS were scarce (3.54%). The proportion of spines with multiple synapses did not vary across layers (KW, *p* = 0.95).

**Fig. 4:**
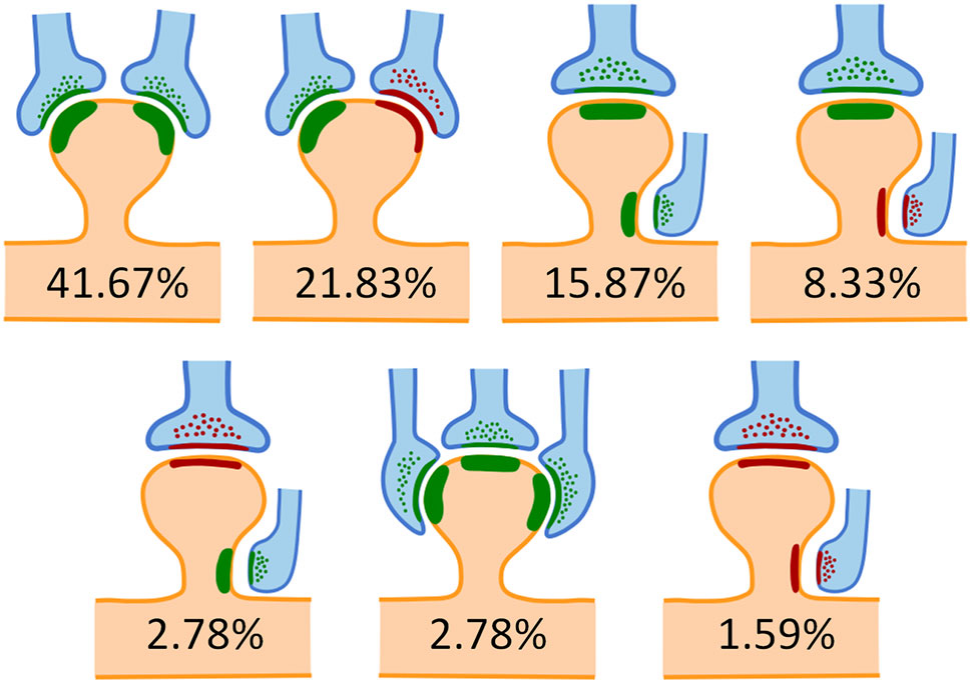
Schematic representation of the different locations of multiple synapses on the head and neck of spines. Asymmetric synapses have been represented in *green* and symmetric synapses in *red*. Percentages indicate the relative frequency of each case with respect to the total number of spines establishing multiple synapses. Other combinations were rarely found (about 5% of all cases) and have not been represented

Regarding the location of synapses on the head or neck of the spine, when spines formed only one synapse, in the vast majority of cases (97.54%), it was an AS located on the head of the spine. In 1.09% of the cases, it was an AS located on the neck, 1.14% corresponded to SS on the head and just 0.23% were SS located on the neck. When considering spines with multiple synapses, there were different combinations of synapses on the head and neck (Fig. 4) and the presence of synapses on the spine neck was much more common. In fact, the proportion of AS on the neck rose to 11.85% (that is, about ten times higher than in spines with single synapses), and the proportion of SS on the neck rose to 5.22% (about 25 times higher than in single synapses).

## Discussion

We have obtained accurate estimations of the proportions of AS and SS and their preferred dendritic targets in the neuropil of each cortical layer, based on a sample of more than 6100 synapses that have been segmented and visualized in three dimensions. The main findings are fivefold. First, AS outnumber SS approximately 9:1 and axospinous synapses outnumber synapses on shafts approximately 8:2. Second, most synapses in the neuropil are axospinous AS (75.54%), followed by AS established on dendritic shafts (14.73%), SS on dendritic shafts (7.08%) and axospinous SS (2.65%). Third, more than 90% of synapses are established on spiny dendrites. Fourth, both AS and SS are more numerous in layer IV and less numerous in layer VI, whereas no significant differences are found between the remaining layers. Fifth, less than 6% of spines establish more than one synapse.

### Sources of synapses

The source of the vast majority of excitatory axon terminals are pyramidal cells, and spines are the main postsynaptic targets of these terminals. There seem to be subpopulations of pyramidal cells whose local axonal arborization selectively form higher proportions of synapses on spines or dendritic shafts. For example, in the cat visual cortex, pyramidal cells in layers II/III and V predominantly established synapses on spines, while layer VI pyramidal cells that project to layer IV mainly established synapses on the shafts (Kisvarday et al. 1986; Somogyi et al. 1998). In our study, we found that the proportion of AS established on spines does not vary across layers—it ranges from approximately 80–90%, which is in line with previous findings in the rat visual cortex (Larkman 1991). However, both the local axon collaterals of the pyramidal cells located within a given region and the axons of pyramidal cells located in other cortical areas (corticocortical connections) contribute to this synaptic population. Since the pattern of corticocortical connectivity (i.e., target cortical areas and layers, and density of axon terminals) varies depending on the species, cortical area and layers under consideration (see (Felleman and Van Essen 1991; Markov et al. 2011) for reviews), the proportion of synapses originating from local or distant pyramidal cell axons should be examined in particular cortical areas and species.

Another major source of AS are thalamocortical afferent fibers and spines are also the main target. The majority of pyramidal cells have significant parts of their dendritic fields in the layers where thalamocortical axons terminate, but there is great variability in the number and proportion of thalamic synapses that different pyramidal cells receive. For example, White and Keller 1989 have shown that in the mouse somatosensory cortex, pyramidal cells projecting to ipsilateral cortical areas, to the thalamus and to the striatum display a characteristic proportion of their layer IV dendritic synapses from thalamocortical axon terminals: corticothalamic cells receive the greatest number of thalamocortical synapses (13.21 ± 5.06% of all axospinous synapses), corticocortical cells receive the next highest number (4.02 ± 2.14%), and corticostriatal the least (0.55 ± 0.15%). Thus, further studies are needed to determine the different proportions of synapses on spines and dendritic shafts in relation to the local, cortical or subcortical origin of the parent axons in the rat somatosensory cortex.

Regarding the origin of SS, the axons of GABAergic interneurons are the major source, and different types of GABAergic neurons form synapses preferentially with particular regions of pyramidal cells or with other interneurons (DeFelipe and Fariñas 1992; Freund and Buzsáki 1996; Houser et al. 1984; Jones 1993; Somogyi et al. 1998). Therefore, not all interneurons contribute equally to the SS found in the neuropil. Several types of interneurons, including Martinotti cells, basket cells and double bouquet cells target spines of pyramidal cells (e.g., (DeFelipe et al. 1990; DeFelipe et al. 1989; Kawaguchi and Kubota 1998; Kisvarday et al. 1985; Somogyi and Cowey 1981; Wang et al. 2004). Therefore, although the proportion of SS synapses on spines is relatively low, they represent an important component of the GABAergic synaptic circuits (Kubota et al. 2015). Finally, the spines establishing SS are likely to be strategically located in the dendritic arbor of the pyramidal cells. For example, Kubota et al. 2007 found that a large proportion of axons forming SS were on spines that were also innervated by thalamocortical axons. Furthermore, double bouquet cells do not appear to form axospinous synapses with apical dendrites, but with basal dendrites and oblique branches of the apical dendrites (DeFelipe et al. 1989, 1990; Somogyi and Cowey 1981), whereas certain types of basket cells (Kisvarday et al. 1987; Somogyi et al. 1983) form numerous synapses with apical dendrites.

### Proportion of AS and SS

The ratio of AS to SS that we found in the neuropil of the somatosensory cortex of the rat at P14 is similar to previous findings in other species and cortical regions using transmission electron microscopy, in which a percentage of asymmetric and symmetric synapses between 80–95% and 5–20%, respectively were reported (Beaulieu and Colonnier 1985; Braitenberg and Schüz 1998; DeFelipe 2011; DeFelipe and Fariñas 1992; Micheva and Beaulieu 1996; Schüz and Palm 1989). Furthermore, we have not found any statistically significant differences in the proportions of AS and SS in different cortical layers, or between supragranular and infragranular layers. In our samples, the highest proportion of SS was found in layer IV. This is in line with data described by Micheva and Beaulieu 1996 who showed that, from P15 onwards, layer IV of the rat barrel field cortex has a higher percentage of SS than the rest of the layers. However, the differences we have found in the somatosensory cortex are small between layers IV, I and VI, and they were not significant in any of the cases. Thus, it seems clear that there are proportionally more excitatory synapses than inhibitory synapses in all cortical layers and areas. The functional significance of this synaptic organization remains to be elucidated.

### Density of synapses

**Fig. 5:**
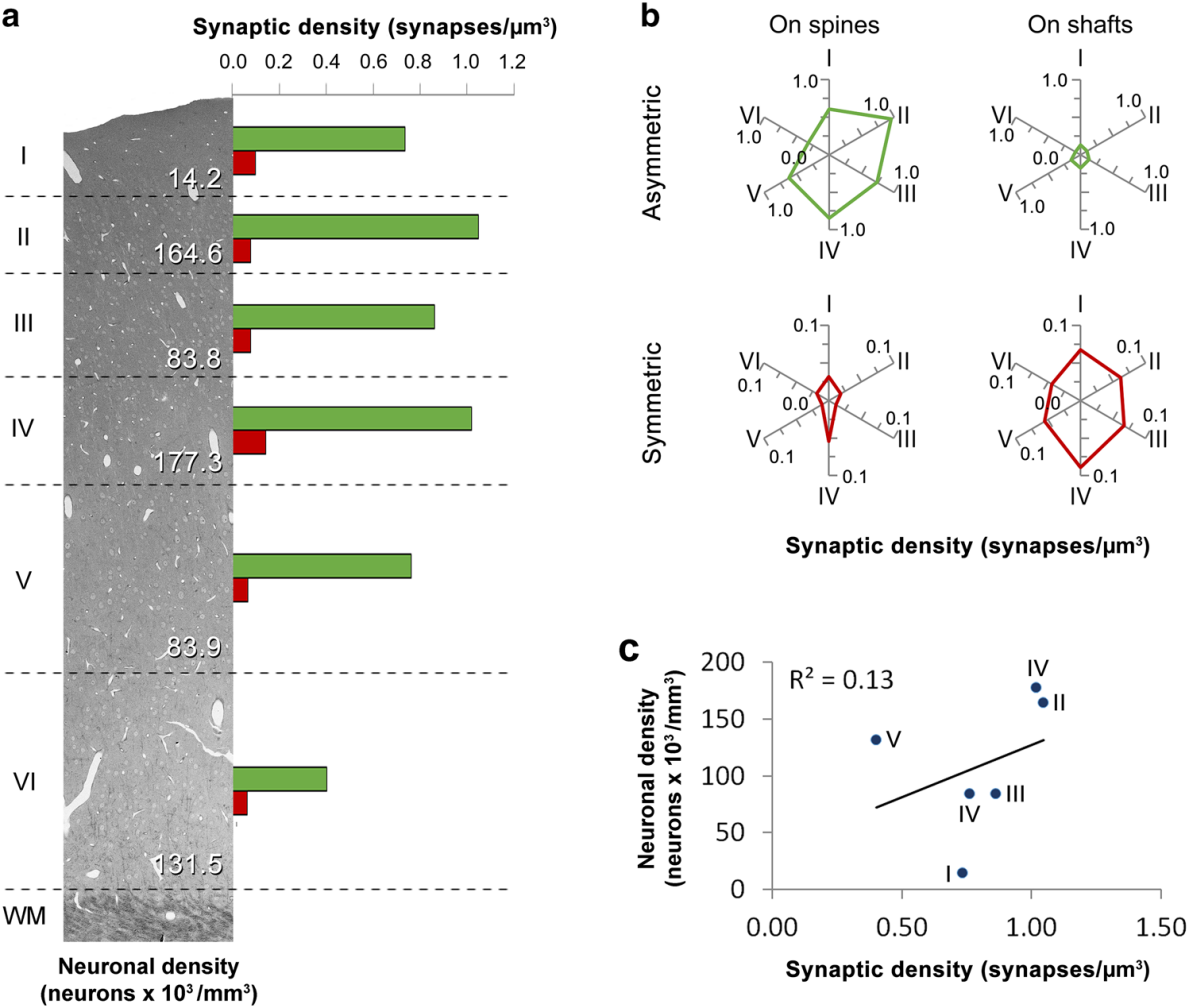
Schematic summary of the results. **a** Neuronal and synaptic densities in the six layers of the somatosensory cortex. The image of the cerebral cortex was taken using the secondary electron imaging mode of the scanning electron microscope. Synaptic densities of AS are represented as *green bars* and SS as *red bars*. *WM*—white matter. The neuronal density data have been taken from (Markram et al. 2015) **b** synaptic densities of AS (*green*) and SS (*red*) on spines and dendritic shafts in the six layers of the cortex. **c** There is no relation between the density of neurons and the density of synapses across layers (*R* ^2^ = 0.13)

Densities of synapses vary across layers, with layers II and IV being the ones with the highest densities. Interestingly, layers II and IV are the layers with the highest density of neurons (Fig. 5); (Markram et al. 2015). However, in layer I, the density of synapses is relatively high but the density of neurons is low, whereas in layer VI the density of synapses is the lowest but the density of neurons is higher than in layers I, III and V. Thus, there does not seem to be a relationship between the density of neurons and the density of synapses. When studying the location of these synapses, we found that there are more AS synapses located on spines in layers II and IV when compared to layer VI, but AS located on dendritic shafts are denser only in layer IV, with layer II and VI having the lowest densities. Therefore, layer II has the highest density of AS synapses located on spines and the lowest located on shafts. When considering SS synapses located on spines, layer IV has the highest density when compared to layers II and V, and there are no differences in the density of SS on shafts across layers.

### Postsynaptic targets

The proportions of postsynaptic targets of synapses in the neuropil have been previously described in the visual cortex of the adult cat (Beaulieu and Colonnier 1985). This study showed that of all synapses, 66.4% were AS located on spines, 17.6% were AS located on dendritic shafts, 5.3% were SS located on spines and 10.6% were SS located on shafts. A study on the distribution of synapses in the rat barrel cortex showed that, at the age of P15, AS are mainly located on spines (82%), followed by dendritic shafts (17%), with less than 1% of the AS on somas (Micheva and Beaulieu 1996). Regarding SS, they are mainly located on dendritic shafts (54%), followed by spines (39%), with just 8% located on the soma. They also reported that, at this age, 15% of the synapses are SS. In the present study, we have found in the rat at P14 that in the neuropil 75.85% of the synapses are AS located on spines, 14.73% are AS located on dendritic shafts, 2.65% are SS located on spines and 7.08% are SS located on shafts. Therefore, in all cases, axospinous AS predominate followed by AS established on dendritic shafts, SS on dendritic shafts and axospinous SS. The different percentages may be due to the different species, cortical areas and ages used (e.g., see (DeFelipe 2011; DeFelipe et al. 1999). It is clear that the preferred targets of AS are spines, and the preferred target of SS are dendritic shafts. Moreover, our statistical analysis indicates that the proportion of SS on dendritic shafts is about 2.70–5.75 times higher than the proportion that would be expected if they were distributed at random between spines and shafts, whereas the proportion of AS that established axospinous synapses was only 1.05–1.08 times higher than would be expected by chance (See Table 2 and Supplementary Table 2 in Online Resource 1). This kind of analysis is based on 2 × 2 contingency tables showing the number of AS and SS against their postsynaptic targets (spines and shafts) and, as such, it does not make any assumption about the possible underlying mechanisms.

### Number of synapses per spine

Classical descriptions of synapses state that one spine establishes one synapse; however, some spines can have multiple synapses (Jones and Powell 1969; Popov et al. 2005). Recently, it has been proposed that there is a link between long-term memory and the formation of multiple synapses when functional strengthening of existing synapses is impaired (Giese et al. 2015; Radwanska et al. 2011). Here, we show that around 6% of spines have two or more synapses, which is a higher proportion than has been reported in previous studies in the hippocampus and neocortex (less than 1%) (Bosch et al. 2015; Petrak et al. 2005; Radwanska et al. 2011). The proportion of multiple synapses on the same spine does not vary across layers. As described above, when spines establish only one synapse, this is excitatory in more than 98% of cases; inhibitory synapses are very rare. However, when spines form multiple synapses, the ratio of AS to SS is approximately 8:2. Therefore, inhibitory synapses on spines occur more frequently when multiple synapses are established. Synapses on the neck of the spine also occur more frequently when multiple synapses are established on the same spine. This could be due to the lack of space on the head of the spine to form multiple synapses, but other molecular and functional factors may be involved, since the proportion of SS and AS on the neck are different. In fact, the proportion of SS and AS on the neck of the spine is about 25 and 10 times higher in spines with multiple synapses, respectively, when compared with single-synapse spines.

In the present study, we have not addressed the issue of the proportion of spines that lack synapses. Rather, we have identified synaptic junctions and then identified the postsynaptic elements. Therefore, no structure lacking synapses has been included in our analysis. Previous studies in the adult mouse neocortex have shown that the proportion of non-synaptic spines is below 4% (Arellano et al. 2007); see also (Bosch et al. 2015; Petrak et al. 2005; Radwanska et al. 2011), but further research is necessary to ascertain whether this low proportion is similar in the juvenile somatosensory cortex.

### Synapses on spiny and smooth dendrites

Most synapses are established in the neuropil (Alonso-Nanclares et al. 2008), which is composed of axons, dendrites and glial processes. The dendritic processes of the neuropil can be spiny or aspiny. In all layers, except layer IV, the origin of spiny dendrites are pyramidal cells—although the dendrites of some GABAergic interneurons are sparsely spiny, there is only a small population of these dendrites in the neuropil. Layer IV includes a mixed population of spiny cell types: layer IV pyramids, star pyramids and spiny stellates. Since pyramidal cells are the most abundant type of cortical neuron (estimated at 70–80% of the total population), it can be assumed that spiny dendrites are the most common type of dendrite in the neuropil. This has been confirmed recently in the 1500 µm^3^ of cortical tissue reconstructed by Kasthuri et al. (2015) who found that 92% of the dendrites were spiny. In general, aspiny dendrites originate from GABAergic interneurons. However, the proximal portion of apical and basal dendrites of pyramidal neurons does not contain spines; reviewed in (DeFelipe 2015). Despite the fact that, in the present study, the sampling of the neuropil was carried out at a distance from the somata of pyramidal cells, we cannot rule out the possibility that some aspiny dendrites correspond to proximal dendrites of adjacent pyramidal cells (stacks containing blood vessels, cell somata or main shafts of apical and basal dendrites in the immediate vicinity of the soma were not used in this study).

In the present study, it is clear that most synapses are established on spiny neurons not only because the vast majority of synapses (78.20%) are established on spines, but also because synapses established on the shafts of spiny dendrites (12.86% of all synapses) outnumber synapses established on aspiny dendritic segments (8.94% of all synapses). Thus, we can conclude that the percentage of synapses on spiny dendrites is at least 91.06%.

In summary, this study provides a large quantitative electron microscopic dataset that will contribute not only to the knowledge of the cortical ultrastructure, but also towards defining the connectivity patterns through all layers of the juvenile somatosensory cortex.

## Electronic supplementary material

Below is the link to the electronic supplementary material.

Supplementary material 1 (PDF 336 kb)

Supplementary material 2 (AVI 101828 kb)
