# Supplementary material for: Volume electron microscopy of the distribution of synapses in the neuropil of the juvenile rat somatosensory cortex
Source: Brain Struct Funct. 2017 Jul 18;223(1):77–90. doi: 10.1007/s00429-017-1470-7 (PMC5772167; doi:10.1007/s00429-017-1470-7)
Supplement: Supplementary file 1 — Supplementary material 1 (PDF 336 kb) [file 429_2017_1470_MOESM1_ESM.pdf]

Volume electron microscopy of the distribution of synapses in the juvenile rat somatosensory cortex.

Andrea Santuy, Jose-Rodrigo Rodriguez, Javier DeFelipe, Angel Merchan-Perez

Supplementary Table1

Layer	Sample	Animal	No. of sections	Resolution (nm/pixel)	Total volume (μm^3)	CF volume (μm^3)	No. of AS	No. of SS	No. of all synapses	AS ($syn/\mu m^3$)	SS ($syn/\mu m^3$)	AS+SS ($syn/\mu m^3$)
I	1	w33	267	3.69	312.93	210.62	161	19	180	0.7644	0.0902	0.8546
	2	w35	301	3.72	358.54	177.20	111	17	128	0.6264	0.0959	0.7224
	3	w31	202	3.72	240.61	167.38	136	19	155	0.8125	0.1135	0.9260
II	1	w33	263	3.69	308.24	224.35	211	19	230	0.9405	0.0847	1.0252
	2	w35	189	3.72	225.13	139.51	117	10	127	0.8387	0.0717	0.9103
	3	w35	237	3.72	282.30	149.03	195	11	206	1.3085	0.0738	1.3823
	4	w31	239	3.72	284.68	213.91	239	18	257	1.1173	0.0841	1.2014
III	1	w31	223	3.69	261.36	149.13	135	12	147	0.9053	0.0805	0.9857
	2	w31	210	3.69	246.12	157.15	101	8	109	0.6427	0.0509	0.6936
	3	w33	214	3.69	250.81	186.45	158	15	173	0.8474	0.0805	0.9279
	4	w33	213	3.69	249.64	176.44	157	21	178	0.8898	0.1190	1.0088
	5	w33	324	3.69	379.73	176.28	148	19	167	0.8396	0.1078	0.9474
	6	w33	219	3.69	256.67	175.55	156	9	165	0.8886	0.0513	0.9399
	7	w33	219	3.69	256.67	191.28	171	18	189	0.8940	0.0941	0.9881
	8	w35	363	3.72	432.39	247.58	186	12	198	0.7513	0.0485	0.7997
	9	w35	247	3.72	294.21	178.40	191	10	201	1.0706	0.0561	1.1267
	10	w35	258	3.72	307.32	165.06	152	16	168	0.9209	0.0969	1.0178
IV	1	w33	235	3.69	275.42	154.59	148	24	172	0.9574	0.1552	1.1126
	2	w35	211	3.72	251.33	158.38	190	15	205	1.1996	0.0947	1.2943
	3	w35	224	3.72	266.82	183.65	151	17	168	0.8222	0.0926	0.9148
	4	w35	253	3.72	301.36	140.63	143	35	178	1.0168	0.2489	1.2657
	5	w35	263	3.72	313.27	123.81	144	18	162	1.1631	0.1454	1.3084
V	1	w33	319	3.69	373.87	165.62	108	9	117	0.6521	0.0543	0.7064
	2	w33	282	3.69	330.51	218.01	181	17	198	0.8302	0.0780	0.9082
	3	w33	310	3.69	363.32	207.95	162	13	175	0.7790	0.0625	0.8416
VI	1	w33	253	3.69	296.52	185.32	84	8	92	0.4533	0.0432	0.4964
	2	w35	285	3.72	339.48	183.55	70	15	85	0.3814	0.0817	0.4631
	3	w31	270	3.72	321.61	179.97	88	14	102	0.4890	0.0778	0.5668
	4	w31	292	4.50	508.96	280.09	92	15	107	0.3285	0.0536	0.3820
Totals			Layer I		912.07	555.19	408	55	463	0.7349	0.0991	0.8339
			Layer II		1100.35	726.80	762	58	820	1.0484	0.0798	1.1282
			Layer III		2934.92	1803.32	1555	140	1695	0.8623	0.0776	0.9399
			Layer IV		1408.20	761.07	776	109	885	1.0196	0.1432	1.1628
			Layer V		1067.70	591.58	451	39	490	0.7624	0.0659	0.8283
			Layer VI		1466.57	828.92	334	52	386	0.4029	0.0627	0.4657

Supplementary Table2

Layer I

		Type of postsynaptic target		
		Spine	Shaft	Totals
Type of synapse	AS	**432** (398.9)	**92** (125.1)	524
	SS	**24** (57.1)	**51** (17.9)	75
	Totals	456	143	599

Layer II

		Type of postsynaptic target		
		Spine	Shaft	Totals
Type of synapse	AS	**825** (788.65)	**86** (122.35)	911
	SS	**13** (49.35)	**44** (7.65)	57
	Totals	838	130	968

Layer III

		Type of postsynaptic target		
		Spine	Shaft	Totals
Type of synapse	AS	**1714** (1602.72)	**300** (411.28)	2014
	SS	**24** (135.28)	**146** (34.72)	170
	Totals	1738	446	2184

Layer IV

		Type of postsynaptic target		
		Spine	Shaft	Totals
Type of synapse	AS	**885** (823.98)	**186** (247.02)	1071
	SS	**59** (120.02)	**97** (35.98)	156
	Totals	944	283	1227

Layer V

		Type of postsynaptic target		
		Spine	Shaft	Totals
Type of synapse	AS	**487** (453.66)	**113** (146.34)	600
	SS	**9** (42.34)	**47** (13.66)	56
	Totals	496	160	656

Layer VI

		Type of postsynaptic target		
		Spine	Shaft	Totals
Type of synapse	AS	**391** (364.38)	**99** (125.62)	490
	SS	**18** (44.62)	**42** (15.38)	60
	Totals	409	141	550

Supplementary Table 1. Densities of asymmetric and symmetric synapses in the six cortical layers. Cortical layer (I–VI), number of samples per layer and animal ID are indicated in the three leftmost columns. Image acquisition details such as the number of sections per sample and image resolution are shown next. In all cases image sizes were 2048 x 1536 pixels and section thickness was 20 nm. The rest of the columns indicate the total volume of each image stack corrected for tissue shrinkage, the volume of the unbiased counting frame (CF volume), counts of asymmetric (AS) and symmetric (SS) synapses, and density of AS, SS and all synaptic junctions per sample.

Supplementary Table 2. Contingency tables showing the type of synapse against the type of postsynaptic target in all cortical layers. The observed counts of synapses in each subcategory were taken from Table 1 and are shown in bold. The expected counts (in parentheses) are calculated from the marginal totals assuming the null hypothesis that there is no association between the type of synapse and the type of postsynaptic target. Chi-square tests of association were applied to these tables indicating that the null hypothesis must be rejected in all cases ($p<0.0001$).
